# The Experience of Couples in the Process of Treatment of Pathological Gambling: Couple vs. Individual Therapy

**DOI:** 10.3389/fpsyg.2017.02344

**Published:** 2018-01-24

**Authors:** Joël Tremblay, Magali Dufour, Karine Bertrand, Nadine Blanchette-Martin, Francine Ferland, Annie-Claude Savard, Marianne Saint-Jacques, Mélissa Côté

**Affiliations:** ^1^Psychoeducation Department, Université du Québec à Trois-Rivières, Québec, QC, Canada; ^2^Addiction Program, Medicine and Health Sciences Faculty, Université de Sherbrooke, Québec, QC, Canada; ^3^Research Service in Addiction, Centre Intégré Universitaire de Santé et de Services Sociaux de la Capitale-Nationale, Centre Intégré de Santé et Services Sociaux de Chaudière-Appalaches, Québec, QC, Canada; ^4^School of Social Work and Criminology, Université Laval, Québec, QC, Canada

**Keywords:** pathological gambling, gambler, treatment, couple treatment, couple

## Abstract

**Context:** Couple treatment for pathological gambling is an innovative strategy. There are some results supporting its potential effectiveness, but little is known about the subjective experiences of the participants.

**Objective:** The aim of this article is to document the experiences of gamblers and their partners participating in one of two treatments, namely individual or couple.

**Method:** In a study aiming to evaluate the efficacy of the Integrative Couple Treatment for Pathological Gambling (ICT-PG), couples who were entering specialized treatment for the addiction of one member who was a pathological gambler were randomly assigned to individual or ICT-PG. Nine months after their admission to treatment, gamblers and partners (*n* = 21 couples; *n* = 13 ICT-PG; *n* = 8 individual treatment) were interviewed in semi-structured interviews. A sequenced thematization method was used to extract the major themes.

**Results:** This study highlighted five major themes in the therapeutic process noted by the gamblers and their partners mainly after the couple treatment but also partly through the individual therapy. These were: (1) the gamblers' anxiety about having to reveal their gambling problems in couple therapy; (2) the wish to develop a mutually beneficial understanding of gambling and its effects on the partners in the two types of treatments; (3) the transformation of negative attributions through a more effective intra-couple communication fostered by the couple therapy; (4) the partners' contribution to changes in gambling behavior and prevention of relapses, which were both better supported in couple therapy; and (5) the interpersonal nature of gambling and its connections with the couples' relationship. However, gamblers who were in individual treatment were more likely to mention that their partners' involvement was not necessary. Participants likewise made a few recommendations about the conditions underlying the choice of one treatment method or the other.

**Discussion:** Participants reported satisfaction with both treatment models, but their experience was more positive in couple treatment. Complementary benefits emerged from each form of treatment, which points to future treatments involving both types. Future research should explore both the couple processes associated with attempts to stop pathological gambling and the various ways of involving partners in the gamblers' treatment.

## Introduction

The prevalence of adults with gambling problems around the world is estimated to be 2.3% (Williams et al., 2012). This difficulty in controlling one's gambling habits leads to considerable negative consequences for pathological gamblers (PG) (Shaffer and Korn, 2002; Lorains et al., 2011), but also for their family members, and in particular for their partners^1^ (Ciarrocchi and Reinert, 1993; Kourgiantakis et al., 2013). Partners report high levels of psychological distress, feelings of anger, fear, loss of security (Dickson-Swift et al., 2005; Kalischuk et al., 2006; Kourgiantakis et al., 2013). They also report many physical symptoms such as headaches, insomnia, stomach problems (Lorenz and Shuttlesworth, 1983; Lorenz and Yaffee, 1988; Dickson-Swift et al., 2005). Gambling habits also entail considerable financial burden, which the partner often has to bear (Dickson-Swift et al., 2005; Hing et al., 2013; Mathews and Volberg, 2013). Finally, the couple is affected considerably, in several respects: less dyadic functioning, sexual difficulties (Trudel et al., 2008), presence of conflicts (Tepperman and Korn, 2006; Dowling et al., 2009; Kalischuk, 2010) and communication problems (Lee and Rovers, 2008; Trudel et al., 2008). Moreover, the presence of lies is a common reality in the interaction with a PG (Dickson-Swift et al., 2005; Patford, 2009; Downs and Woolrych, 2010; Hing et al., 2013), leading to a loss of confidence and a sense of betrayal felt by the partner (Dickson-Swift et al., 2005; Hing et al., 2013).

Despite the many consequences for gamblers, only a small proportion of PG (3–19%) ask for formal help, begin treatment, and partake in meetings of Gamblers Anonymous, whether this be in the last year (Slutske, 2006; Slutske et al., 2009) or during their lives (Suurvali et al., 2008). What is more, many gamblers quickly drop out of the therapy process after beginning treatment (Melville et al., 2007; Giroux et al., 2015).

Even though there are numerous negative consequences for the partners, the current treatment models, often based on individual intervention, make little or no room for them in the rehabilitation of gamblers. Several authors however, particularly in the field of drug and alcohol abuse, point to the relevance of integrating family members in the treatment (Bertrand et al., 2008; Tremblay et al., 2009). Indeed, studies in the addiction field have shown that the involvement of partners in treatment improves treatment entry (Manuel et al., 2012; O'Farrell and Clements, 2012), enhances retention, the achievement and maintenance of sobriety, and relationship satisfaction, and reduces domestic and family violence (McComb et al., 2009; Klostermann et al., 2011; McCrady, 2012; O'Farrell and Clements, 2012).

As of yet, there is little data allowing us to understand the role that partners play in the rehabilitation of pathological gamblers. Nonetheless, the few studies that have examined the role of family members have also mentioned the added effectiveness in terms of the treatment retention rate when the partners participate in the pathological gamblers' therapy (Ingle et al., 2008; McComb et al., 2009). It would seem, moreover, that a better access to social support is associated with a reduced probability of relapse in pathological gamblers (Oei and Gordon, 2008) and an improved outcome (Petry and Weiss, 2009). University of Calgary team adapted the Community Reinforcement Approach and Family Training (CRAFT) model (Meyers et al., 1996) for pathological gambler partners (Makarchuk et al., 2002; Hodgins et al., 2007; Nayoski and Hodgins, 2016). They reported partners well-being improvement and a higher probability of getting the PG into treatment. Some clinical articles have likewise reported the value of integrating the partners of these gamblers in their treatment without having objectively documented this (Steinberg, 1993). In reaction to the predominance of individual treatments (Kourgiantakis et al., 2013), we developed (Bertrand et al., 2008; Tremblay et al., 2015) like a few other research teams, couple therapy models to try and better understand the influence of partners (Ciarrocchi, 2002; Lee, 2002; Lee and Rovers, 2008). These few studies, limited due to the number of participants, seem to indicate that couple therapy improves the treatment retention of pathological gamblers, as well as being more effective than waiting list controls (Lee and Rovers, 2008; Lee and Awosoga, 2015). No studies have, as of yet, compared couple treatment with individual or group treatment.

While the initial quantitative research results lead us to think that the partners' integration has an objective and positive impact on treatment success, no study has yet explored the gamblers' and their partners' viewpoints about the perceived benefits of this form of treatment. More knowledge of these couples' perceptions would allow us however to better understand the processes involved in treatment and to estimate, from a perceptual view, the dimensions and relational underpinnings that should be targeted in a couple-based treatment model. The qualitative data, as limited as it is, points to the presence of various deficits in the relationships of couples where one is a pathological gambler (e.g., communication difficulties, little intimacy, little time spent together, over- vs. under-involvement in family responsibilities) (Lee, 2014). The consequences of gambling behavior seem to aggravate these deficits by reviving old wounds, accentuating emotional reactivity, and creating a relational imbalance through an increase in shame and guilt on the one hand and control and hypervigilance on the other (Lee, 2014). The rehabilitation of gamblers who live with their partners is part of a relationship process that takes each person's life into account, establishing a climate of trust (that repairs the damage caused by the lies and betrayal) and employing constructive communication (as opposed to attacks) that permit, among other things, a discussion about the triggers that provoke the gamblers' cravings (Lee, 2015).

Current knowledge of the rehabilitation process and, more specifically, of the rehabilitation elements associated with the couple is limited and scarce. Consequently, documenting clients' perceptions of these processes may be prove to be valuable in designing and implementing new treatments, such as couple therapy. The aim of the present study was to document the experiences of the therapy process for pathological gamblers and their intimate partners, who were randomized for individual or couple treatment. The perceptions and opinions of the gamblers and their partners about the treatment they received were then collected.

## Method

The participants were recruited as part of a larger longitudinal research project whose main objective was to evaluate the effectiveness of couple treatment (Integrative Couple Treatment for Pathological Gambling–ICT-JP) (Tremblay et al., 2015) as compared to the usual (individual) form of treatment. To be admissible to the research project, the couple had to be living together for at least 6 months and be 18 years old or over. One of the couple members had to be diagnosed as being a pathological gambler as measured by WHM-CIDI (Kessler and Üstün, 2004) and to have requested help from one of the seven addiction treatment centers in the Province of Quebec participating in the study. Both members of the couple could not have alcohol and drug abuse problems as assessed with the DEBA-Alcohol and Drugs (Tremblay et al., 2016). Income was assessed through a house-based sociodemographic questionnaire providing income categories. Gambling behaviours were recorded as a frequency and money lost, by activity and total. The financial losses du to gambling were transformed into percentages of personal and couple annual income. Psychological distress was evaluated with the IDPESQ, a 29 items scale questioning about anxiety, depression, anger, somatization and cognitive difficulties and largely validated among Quebec population (Préville et al., 1992). The four items version (Sabourin et al., 2005) of the Dyadic Adjustment Scale (Spanier, 1976) was used as a well validated short scale of marital satisfaction.

The participants in the qualitative part of the project were recruited and questioned about their therapy experiences during the evaluation meeting held 9 months after admission into therapy. The number of participants was set when empirical saturation was attained (Pirès, 1997; Creswell, 2013). The data collection was conducted from June 4, 2012 to July 22, 2014^2^.

### Participants

A total of 21 couples, gamblers and partners, were interviewed, of which eight were oriented in individual therapy and 13 in couple therapy. The gamblers oriented in individual treatment received the usual treatment offered in the rehabilitation center in addiction while their partner were offered treatment through the family members services available at the center (mostly individual treatment, helping them to care about themselves but also including a psychoeducational part, helping partners to better understand the gambling disorder). When the couple was oriented in couple modality, the gambler, and his partner met a clinician who applied the ICT-PG (Tremblay et al., 2015). All gamblers and their partner provided a written informed consent. A certain number of participants had access to both treatment models, either because they were in therapy in the previous year before participating in the research project^3^, or because, after the treatment phase of 8–12 meetings, they could ask to have access to the other treatment model if they needed to continue. Three people from the individual therapy and five people from the couple therapy were in this situation.

The gamblers were primarily men (87.5%) and their partners were women (85.7%), except for one couple composed of two women. The mean length of time they had been living together was 13.8 years (Min = 1 year; Max = 71 years; SD = 14.7). The couples' combined annual incomes were distributed over all three levels, with 28.6% having a low income ($40,000 and less), 33.3% moderate ($40,001–$100,000), and 38.1% high (more than $100,000). As regards the 3 months preceding treatment admission, close to two thirds of the gamblers reported having played video lottery terminals (VLTs), 28.6% played various casino games, 14.3% cards (poker), and 14.3% Internet gambling. During the same period, the gamblers had financial losses equivalent to 38.7% (Min = 0%; Max = 229%; SD = 60.78%) of their individual income and 22.1% of the couples' income (Min = 0%; Max = 123%; SD = 34.2%). Almost half of the gamblers (47%) and more than half of the partners (60%) reported being dissatisfied with their couple relationship (DAS-4 ≤ 4). At the psychological level (IDPESQ-29), 43% of the partners and 19% of the gamblers reported having experienced a high level of distress in the 7 days preceding their admission into treatment.

### Qualitative interview

Semi-structured interviews of a mean length of 20.1 min (Min = 10 min; Max = 55 min; SD = 9.7; non significative differences in duration between gamblers and partners and between participants in individual or couple therapy) explored the participants' therapy experiences, the perceived effects of the therapy, the therapy elements that contributed to or hindered change, and the couples' recommendations concerning the therapy. The proposed themes proposed to participants were similar for all (gamblers and partners, couple vs. individual therapy) with minor adaptations to their specific situations. The interviews were conducted by four master students from a counseling field and were held in the treatment centers. The spouses were interviewed separately.

### Qualitative analysis

This paper presents a descriptive and interpretative qualitative study within an existing research initiative, which is a randomized controlled trial aiming to evaluate the efficacy of the ICT-PG. This study is grounded in the interpretative tradition (Giordano, 2003), where individuals make their own interpretations based on their subjective experiences of the world (Brunelle et al., 2015). Specifically, a descriptive phenomenological approach (Giorgi, 1997) guided the development of the interview guide and the analysis process. Descriptive phenomenology focuses on exploring how human being give sense of to this experience and how he describes, perceives this experience (Patton, 2002). At the end of the analysis, the researcher can state that actual experiences gathered from the individuals involved come from their own experiences and not from objective accounts of what truly happened (Giorgi, 1997). Accordingly, we took note of participants' descriptions of their experiences without forcing the meanings of their interpretations into our own categories.

On a technical point of view, sequenced thematization (Paillé and Mucchielli, 2012, 2016) was used in this study. This type of analysis makes it possible to extract themes and sub-themes from the interviews so as to summarize the collected statements (Paillé and Mucchielli, 2012, 2016). To do so, different stages were carried out during the analysis. To begin with, all the audio recordings were integrally transcribed by three research assistants. Themes and subthemes began to emerge after the reading of a few randomly selected interviews (four protocols, i.e., one partner and one gambler in individual therapy, and one partner and one gambler in couple therapy). These were integrated into a coding table created by one of the project researchers together with the research assistants in charge of codifying the interviews. This coding table represented a thematic concept tree with a description specific to each theme and sub-theme so as to ensure that the whole research team had a uniform understanding of the concepts. Subsequently, two research assistants codified the remaining interviews using this coding table. Since two people codified the interviews, three interjudge agreement processes were conducted during the analysis. A total of six interviews were codified by two assistants and the classification differences were discussed so as to ensure coding uniformity. This coding work was conducted under the supervision of the main researcher and used the qualitative data analysis software *QSR International NVivo 9*. Once the whole corpus was codified, the two research assistants created a summary for each of the codified hubs. A comparison of the participants' statements as a function of the treatment modality received by participants was conducted.

## Results

The analysis of the participants' statements drew out several crucial themes from the overall changes that occurred, in the participants' eyes, due to the treatment they received. Most of the themes were shared by the gamblers and their partner in the same treatment modality but many themes differentiated couples in the two modalities (see Table 1). The participants also described the different ways that the treatment enhanced their efforts to make progress in various aspects of their lives.

**Table 1:** Themes reported by participants in function of treatment modality received and status (i.e., gambler vs. partner).

**Themes**	**Individual therapy**		**Couple therapy**	
	**Gambler**	**Partner**	**Gambler**	**Partner**
1. Revealing gambling behaviors to the partner	x		x	
2. The need to develop mutual comprehension and the need for help to attain it				
a) The partner's need to understand the change process	x	x	x	x
b) The need to have discussions about their mutual experiences	x	x	x	x
c) The benefits of having a neutral person present			x	x
d) The practice of communication			x	x
3. Better mutual comprehension improves mutual support				
a) The couple approaches the gambling problem together			x	x
b) No longer reinforce gambling behavior			x	x
c) Gambling behavior interpreted as meanness			x	x
d) Gamblers develop a better understanding of their partners' suffering			x	x
e) The partners help the gamblers to avoid relapses			x	x
f) The couple starts to do enjoyable activities together again			x	x
4. Commitment to and regularity in treatment	x	x	x	x
5. For many, gambling is a relational problem	x	x	x	x
6. In some gamblers' opinion, gambling does not concern the couple	x			
7. Format and structure	x	x		x
8. Conditions favouring one treatment or the other				
a) Conditions favouring individual treatment	x	x		
b) Conditions favouring couple treatment			x

**1. Revealing gambling behaviors to the partner**
  All gamblers noted that one of the delicate tasks of the change process was to be honest about their gambling cravings and behaviours, in particular toward their partner. Some gamblers reported not wanting to reveal everything to their partners, this being all the more true for those for whom lying was a well-established behavior.
  “*Sometimes it's better your girlfriend doesn't know certain things. They're not really lies, they're personal things you don't want her to be aware of.” [5191-Gambler_CoupleTherapy*^4^*]*
  “*When you are an addict, whether it's alcohol, gambling, or drugs, you're a liar too. [So, your partner] she doesn't really know [what you do].” [14331-Gambler_CoupleTherapy]*
  Gamblers in the two types of treatment mentioned this point, emphasizing how individual treatment was better in this regard than the couple format, which did not encourage gamblers to reveal everything to their partners.
**2. The need to develop mutual comprehension and the need for help to attain it**
  As opposed to the preceding theme, several couples emphasized both their need to mutually understand each other and their need for help to achieve this. They had the impression that they lacked the communication skills to talk about the difficulties caused by gambling.
  “*Our ways of expressing ourselves and understanding each other weren't very good. […] He didn't really understand what I was trying to say, and I didn't understand him either.” [16310-Partner_CoupleTherapy]*
  a) The partner's need to understand the change process
    A first demonstration of the necessity to develop mutual comprehension was the need expressed by the partners from both modalities to grasp what was going on in the minds of the gamblers vs. the potential difficulty of the gamblers to talk about their progress in the therapeutic process.
    These needs did not seem to be responded in the individual treatment. Partners from this modality stated that they noted changes in the gamblers during the therapy process but that they did not understand why the latter engaged in excessive gambling behavior. This lack of information led them to be suspicious of the gamblers, to not believe what they said.
    “*I would like to have known why… What was going on in his head. […] I had a hard time believing what he told me. I would have liked [to know] if the meetings were having an impact on him, to reassure me.” [16230-Partner_IndividualTherapy]*
    Furthermore, gamblers in individual treatment reported how difficult it was for them to tell their partners what they had said during the therapy sessions, either because they did not wish to share it or because they had a hard time finding the right words.
    “*When I consulted individually [this gambler had individual treatment during the years before actual project participation], I didn't tell my partner what happened during the meetings. It was already intense enough in the meetings, so I left it there. [At the time I said to myself]: It's my problem, so why involve him.” [3380-Gambler_CoupleTherapy]*
    “*Your partner doesn't know what you're working on to get through it. She's not doing it. You can try and explain it to her after [the individual meetings] at home, but it's not the same.” [16161-Gambler_IndividualTherapy]*
    At the opposite, couple treatment helped partners to better understand underlying gambling motivations and gamblers to talk about their inner experience concerning gambling urges and behaviours.
    “*Gambling is difficult to understand by a partner. […][Couple therapy] helps partners to understand gambler's problem and gamblers to explain gambling behaviours to the partner” [3240-Partner_CoupleTherapy]*
  b) The need to have discussions about their mutual experiences
    Both members of the couple mentioned the need to have time to talk about themselves mutually and respectfully, so as to more clearly express their emotions and ideas and better understand the other person's.
    Couple treatment seemed to genuinely provide a moment for respectful exchanges that were more difficult to have at home. The couple meetings obliged them to stop, listen to each other, and talk about themselves, which did not seem to occur otherwise.
    “*With the therapist, [my husband] doesn't have the choice but to let me finish my sentences […], to listen right to the end. […] At home, he would have listened to half the sentence [and] then filled in the rest.” [3240-Partner_CoupleTherapy]*
    Partners and gamblers in individual therapy likewise reported that they would have liked to have taken part in couple therapy to benefit from the organized exchanges.
    “*I would have liked that [couple treatment] […], it seems more helpful. You are both there, you can hear what the other person has to say […], we could have had a real discussion for once.” [14410-Partner_IndividualTherapy]*
    “*Well, to be honest, we wanted to be together [in couple therapy] because we wanted to understand. But now that we're in individual treatment, I find it a bit… disappointing.” [16230-Partner_IndividualTherapy]*
  c) The benefits of having a neutral person present
    The presence of a therapist with a neutral attitude facilitated constructive communication. The therapist helped the couple to talk honestly about themselves to their partners, but also to listen sincerely. This idea was mentioned only by couple therapy participants.
    “*It's a lot easier with a neutral person, you feel like you have to tell the truth about what you're feeling, about what the other person is putting you through.” [3260-Partner_CoupleTherapy]*
  d) The practice of communication
    The therapist also helped the couple to express what they were going through, to find the words to describe what they were experiencing and feeling, but also to ensure that they listened carefully to each other. Several couples oriented in couple treatment mentioned communication strategies established by the therapists during the meetings, including: the right to speak and the need to listen, rephrase, clarify, elaborate, and use words better suited to a constructive exchange. The mediation skills seemed to help the couples to talk about themselves better, listen better, and thus understand better.
    “*It made me realize […] I should double check on what my wife thinks. […] I mean, did I understand her, [tell her] what I understood, then wait for the answer. Sometimes I think [I've understood and] that she's understood, but perhaps we haven't really understood each other at all.” [14331-Gambler_CoupleTherapy]*
    Some gamblers and partners mentioned how they progressed in their ability to speak about themselves because of the couple treatment. Moreover, talking about oneself in front of one's partner allowed the participants to immediately grasp the reaction of the other and thus improve their mutual understanding.
    “*I'm not a very talkative guy, but here, I have to talk about myself, […] about my feelings. I've never done that before. For sure [couple treatment] helped me to learn how to talk more about myself and be more open-minded.” [16211-Gambler_CoupleTherapy]*
    “*I think you solve more communication problems right away by saying straight out [what you think], face-to-face.” [14371-Gambler_IndividualTherapy_CoupleTherapy]*
**3. Better mutual comprehension improves mutual support**
  The fact of better understanding the other person's psychological experiences often led to an increase in empathy and greater tolerance. All the ideas included in this third global theme were mentioned only by the gamblers and partners oriented in couple treatment.
  a) The couple approaches the gambling problem together
    The partners in couple therapy understood their gambling spouses better and, for some, a discussion began around the theme of gambling and gambling cravings that lasted all week long, thereby creating a place for discussion that extended beyond the treatment meetings.
    It's as if the meetings lasted longer […] because there's one whole hour here, but then there's all those short moments [where we talk] during the week. It's like it's teamwork. [3380-Gambler_CoupleTherapy]
    Some gamblers also had the impression that their partners, because of their physical and emotional proximity, became a more accessible resource than the therapist when they had strong gambling cravings or even relapses. By better understanding the causes of the spouses' gambling behavior, the partners were in a better position to help them.
    “*She had already come for individual treatment. Even though she talked to me about her gambling problems, I think I didn't have the right tools to understand her completely. But coming to couple treatment has given me the tools I needed to understand her, talk about it, and help her so it doesn't start again.” [3381-Partner_CoupleTherapy]*
  b) No longer reinforce gambling behaviour
    The partners who came to the couple meetings reported having learned how to reduce the situations that stimulated their spouses' gambling cravings, while at the same time stating that it was difficult to achieve.
    “*You always have to be careful. I don't buy any scratch and win [tickets]. […] And I'm always watching over people who [might cause a relapse]. But it's not easy to do.” [3240-Partner_CoupleTherapy]*
  c) Gambling behaviour interpreted as meanness
    The partners frequently perceived gambling behavior as a mean and spiteful action by the gamblers, or as an absurd, meaningless behavior.
    “*Because, most of the time, [you say] ‘How can you do that? Come on, that's crazy? You've got a brain, you can stop [gambling].’ It's difficult to understand for a person who doesn't have the problem.” [3240-Partner_CoupleTherapy]*
    A growing, mutual understanding helps them to modify this perception and to see the suffering underlying gambling behavior. Through couple treatment, a better understanding helps the partners not to attack and belittle their gambling spouses so often, and to try to support them in the rehabilitation efforts. The gamblers clearly highlighted this issue and considered that the perception of meanness was erroneous.
    “*She didn't understand gambling at all. She thought everything could be healed, that only crazy people gamble. [Now] she has another perception, [she] understands what happens to me, that I don't go because I feel like gambling all our money. […] Now it's easier for her to support me rather than just shouting and killing me.” [5191-Gambler_CoupleTherapy]*
    “*My wife, she thought I wanted to hurt her*^,^ *but that wasn't it at all. Gambling is stronger than I am, I go even though I know I shouldn't.” [16311-Gambler_CoupleTherapy]*
  d) Gamblers develop a better understanding of their partners' suffering
    The gamblers did not always realize the impact of their gambling behavior on their partners. The couples' therapy meetings helped to increase this understanding, which relieved the partners.
    “*He [my gambling spouse] looked at me […] and said ‘I didn't know that it hurt you so much and that you were that scared.’ It was as if he had never realized.” [3260-Partner_CoupleTherapy]*
  e) The partners help the gamblers to avoid relapses
    The partners helped to create an environment in which their spouses' gambling cravings and behaviors were not encouraged. They did this by remaining vigilant about preventing potential gambling stimulations, including such things as time spent with gambling friends. The gamblers sometimes mentioned how much they needed their partners' support to maintain what they had achieved.
    “*I have a disease that will always be there. She knows that the support she gives me is very important [which means that] now I only gamble very rarely or almost never. Now she understands her importance to me, and me to her too. Now we're important for each other.” [14371-Gambler_IndividualTherapy_CoupleTherapy]*
  f) The couple starts to do enjoyable activities together again
    Several participants in the couple therapy mentioned that they had stopped participating in simple couple activities (e.g., going to the movies, holding hands) or more elaborate ones (e.g., travel), but that they had started doing them again, realizing the pleasure it added to their relationship.
    “*We've been together for 24 years and we've never held hands [saying] ‘I love you’ and things like that. So now we've learned to do it.” [3180-Gambler_CoupleTherapy]*
**4. Commitment to and regularity in treatment**
  The couples in both treatments raised the issue of the gamblers' motivation, particularly the need to help them go to treatment regularly. The gamblers' motivation to reduce their gambling habits and thus to continue attending the meetings had to be encouraged by the partners, a fact that was noted by both the gamblers and their partners.
  Couple therapy is perceived as more helpful than individual one to sustain regularity in treatment. Several of the gamblers selected for couple treatment mentioned that, if it had not been for the presence of their partners, they would not have continued the treatment. The fact of making a commitment to their partners and feeling supported by them was of considerable importance. Partners who participated in the couple treatment were of the same opinion.
  “*I don't know if I would have made it to the end. Sometimes it takes a little kick in the butt. I don't know if I would have had the motivation to come every time, it's easier to do it together. […] Sure I'm the one who has the problem, [but with] someone to support you all the time, it's a bit easier.” [5191-Gambler_CoupleTherapy]*
  “*I also made a commitment to my partner in that [couple] therapy. It wasn't just a promise to myself, I think it meant more to me to have to commit [to] both of us to no longer gamble.” [3380-Gambler_CoupleTherapy_IndividualTherapy]*
  Similarly, the partners of the gamblers who were in individual treatment considered that their gambling spouse would have gone more regularly to treatment meetings and thus would have made better progression if they, the partners, had gone to the same treatment meetings.
  “*If we had been in couple treatment together, it would have certainly lasted longer. He would probably have gone right to the end [of the treatment]. Even if I had to drag him on a leash [to the meetings].” [14280-Partner_IndividualTherapy]*
**5. For many, gambling is a relational problem**
  Several couples in both treatments considered that gambling problems were intertwined with the couples' relationship and that it was therefore necessary to discuss everything during the couple meetings. For these participants, opting for couple treatment was an obvious choice, responding more directly and effectively to the gambling problem and its relationship dimension.
  “*The couple is the problem. If you gamble […the problem] is in the family too. You don't have any more [money], you don't spend anymore [for the family], you keep it up, you always go out alone. I think couple therapy is better for bringing the two together, we'll solve the problem together.” [16271-Gambler_CoupleTherapy]*
  “*Yeah, it's important. Sure, I'd recommend [couple therapy], because I don't think it can work all alone. It can't, it's not the same thing. I can't say to my spouse, “Fix your problem [yourself], [because] it's our problem. We live together for better or for worse, so it's our problem.” [3260-Partner_CoupleTherapy]*
  Furthermore, these couples reported that the gambling problems caused the partners considerable suffering and that couple treatment made it possible to help both members of the couple.
  “*I think all couples would be better off doing the couple therapy. Because I think the person living with someone who has a [gambling] problem suffers as much as the gambler. You help two people in difficulty. Two birds with one stone.” [16311-Gambler_CoupleTherapy]*
**6. In some gamblers' opinion, gambling does not concern the couple**
  Inversely, some gamblers oriented in individual treatment considered that they were much better off in individual treatment, believing that their partners would have wasted their time in these meetings. They did not want to talk to their partners about their personal difficulties. Couple therapy would only have been useful for dealing with relational conflicts. No gamblers in couple therapy expressed this opinion.
  “*[If I was going to couple therapy], we'd really have to come to an agreement about the fact that I need to talk without her being there. […] I think [couple therapy] it's not the place to talk [about more personal difficulties].” [3441-Gambler_IndividualTherapy]*
**7. Format and structure**
  Most of the couples were satisfied with the services received, whether it was the individual or couple therapy. A few people who were selected for individual treatment and subsequently received couple therapy, considered that a combination of the two types of treatment would have been beneficial, beginning with individual meetings and then working with the couple. The partners mentioned that this would have allowed them to speak more openly during the individual treatment without running the risk of hurting the gambling spouses. What is more, when the partners obtained services through the family members services (only for couples randomized to individual therapy), there was a teaching section in the individual therapy where the partners learned about the psychology of gamblers, how pathological gambling develops, and all the various elements that would help them understand their gambling spouses. Subsequently, the couple meetings allowed them to talk about relationship problems.
  I think it's important the partner learns what it means to be a gambler, what his strategies are, everything surrounding gambling. Then she'll be more prepared for couple meetings, it'll be easier to understand what her [gambler] spouse is saying. To be able to say what we really want to say without being afraid of hurting the other person, of pushing him to gamble more. […] Individual treatment followed by couple treatment, I think that would be perfect. Individual is okay, but [at] some point, you absolutely need couple treatment. [14370-Partner_IndividualTherapy_and_CoupleTherapy]
  The gamblers oriented in individual treatment agreed for the most part that it would have been too difficult to begin with couple meetings. They would have had the impression of not being able to speak freely, of feeling “tense.” They thought however that, after having taken the time to straighten out their personal situations, it would have been beneficial to continue with couple meetings.
  “*Begin in individual, work on some things, then after, do some couple therapy. I'd suggest that to lots of people. If I had begun in couple, things would have seized right up. It wouldn't have helped me. If you can, do individual for a while like I did, then after jump into couple therapy. Cause then, you've worked on problems, you've understood some things that you wouldn't have understood [in couple treatment]. Individual helped me to get some of the bad things out, to understand stuff. Then you go to couple treatment and you can go farther.” [3441-Gambler_IndividualTherapy]*
**8. Conditions favouring one treatment or the other**
  The participants wanted to talk about the conditions favouring orientation to individual or conjugal treatment.
  a) Conditions favouring individual treatment
    In situations where the gamblers had great difficulty expressing themselves and where the partners talked a lot and even too much, it might be better to direct the gamblers toward individual treatment, and this in the opinion of one partner who considered she talked too much.
    Furthermore, when gamblers did little to meet the family's needs and invested little in the couples' relationship, their partners felt relieved to know their gambling spouses were consulting individually, as this gave them the impression they had a bit less to carry on their shoulders. The partners felt that they had to overcompensate for their spouses' passiveness. The fact that the gamblers themselves asked for help thus represented a step toward their self-sufficiency and a lightening of the partners' own excessive load in family tasks and chores.
    “*That [the fact he is going to individual therapy] has lightened my load. Because he depends a lot on me to get through life. So, the fact that I didn't have access [to his therapy] let me relax. I had one less worry. Somebody else was taking care of him. It took a load off my shoulders, gave me one less thing to take care of in his rehabilitation process. Before I had all the responsibilities because he refused to take any. It's easy to delegate everything to your wife; when you don't take on any responsibilities, you can't be blamed for anything. Now when the telephone rings because this or that happened, I don't worry about it. He has to take care of it.” [14280-Partner_IndividualTherapy]*
    Other couples mentioned that individual treatment allowed them to progress at their own rate by, among other things, adapting the frequency of the meetings to their personal needs.
    In situations where the gamblers had to explore different elements of their childhood or adolescence, it was sometimes advantageous to turn to individual treatment, thereby giving the gamblers all the space they needed to talk about themselves freely.
  b) Conditions favouring couple treatment
    One gambler who took part in the couple treatment mentioned that, to be selected, the couple already had to have a trusting relationship. He reported that his therapeutic progression has led to make connections with his childhood, consequently revealing part of his personal history to his partner. He reported how the wish to create a strong couple relationship was a very helpful tool to embark on pathological gambling treatment as a couple. Other more shy gamblers mentioned that their partners presence made it easier to open up before the therapist. They felt that being alone with the therapist would have made it more difficult to talk about themselves.
    Other gamblers clearly recommended couple treatment when the gamblers wanted to save their couple relationship. Mutual understanding fostered reciprocal support whereas individual therapy risked resulting in a breakup.
    “*If the person doesn't care for his partner, [let him] do it [the treatment] all alone. But if you want your relationship with your wife to survive, I think it's better to involve the other person who's in it. That way, she can understand you better, and then she'll be able to help you. And so will you, you'll understand things too.” [14331-Gambler_CoupleTherapy]*

## Discussion

This study, which to our knowledge is the first to document and compare gamblers and their partners who have received either individual or couple treatment for pathological gambling, helps us to better understand the therapeutic processes in play, as well as to determine the issues involved in choosing between the different types of treatment. Even though the impacts of pathological gambling on couples have been well described in various quantitative studies (Trudel et al., 2008), these couples' experiences in gambling rehabilitation and the therapeutic process have gone largely unexplored. That being said, we must take these experiences into consideration if we are to develop and improve treatment for pathological gambling that takes into account the couples' personal situations and needs.

This study highlighted five major themes in the therapeutic process noted by the gamblers and their partners after the individual or couple treatment, namely: (1) the gamblers' anxiety about having to reveal their gambling problems in couple treatment; (2) the wish to develop a mutually beneficial understanding of gambling and its effects on the partners in the two types of treatment; (3) the transformation of negative attributions through a more effective intra-couple communication fostered by the couple therapy; (4) the partners' contribution to changes in gambling behavior and prevention of relapses, which were both better supported in couple therapy; and (5) the interpersonal nature of gambling and its connections with the couples' relationship. Moreover, the participants made a few recommendations as to the conditions which helped to choose one type of treatment or the other.

The impact of pathological gambling on couples was different according to the viewpoint, namely that of the gambler or partner. Regardless of the treatment received, the gamblers spoke of the difficulty of talking about themselves in front of their partner, and even more so of talking about their gambling behavior. Indeed, numerous authors have reported how gamblers constantly lie to their partners (Lorenz and Yaffee, 1989; Dickson-Swift et al., 2005). Gambling behavior is relatively easy to hide given that it leaves no physiological traces (McComb et al., 2009). Gamblers are aware of the potential negative impact of their deception on the quality of the couple relationship and are thus afraid of losing their partners' trust and arousing their anger. The gamblers' dishonesty is reported as one of the main causes of tension and conflict in couples' relationships (Blaszczynski et al., 1999; Dickson-Swift et al., 2005). These were probably the issues in the present study that led gamblers' to mention that individual treatment allowed them to reveal more of themselves than did couple treatment. Moving from a period of constantly lying to one's partner to one of telling the truth is certainly not simple and produces considerable apprehension in gamblers. As for the partners, they largely expressed the need to develop better mutual understanding, a need that was shared by the gamblers, and this despite their apprehensions.

On the one hand, the partners, having been confronted for years with the dissimulation of gambling habits, found it difficult to understand the reasons that pushed the gamblers to such extreme behavior. They consequently tried to shed light on the situation. On the other hand, the gamblers reported not knowing how to talk to their spouses about their progress when they were in individual therapy. Other qualitative studies have reported on these communication difficulties (Lee and Rovers, 2008; Trudel et al., 2008), which comprise such patterns as negativity, unproductiveness, blame, avoidance, and withdrawal (Lee, 2002). Participants in couple therapy reported how this treatment type helped them to develop their communication skills, primarily through the presence of a neutral, third person but also through exercises that were practiced during the meetings. Some of the participants in the individual treatment mentioned that they would have liked to try the couple treatment. By mentioning the crucial role of the therapist, the participants were indirectly referring to the concept of the therapeutic alliance. This, in the context of couple therapy, includes the notion of a safe climate created by the therapist as a fundamental element which allows both members of the couple to talk about themselves without fear of being counterattacked (Beck et al., 2006). The results of a meta-analysis found moreover that therapeutic alliance in a couple treatment showed a moderate effect size as to the prediction of the treatment's effectiveness (Friedlander et al., 2011). Given that lying, but also anger and the desire for vengeance are common in couples where one member is a pathological gambler, a safe, neutral climate where each person can be equally heard (Boszormenyi-Nagy et al., 1991) allows people to express themselves and listen to the other person talk about fundamental relationship issues.

Tepperman and Korn (2006) had previously noted that a better couple relationship was associated with greater interest on the part of gamblers to modify their gambling behavior. The results on the opinions of couples who received couple treatment made it possible to better document the processes leading to a higher probability of the gambler changing. These couples reported how better communication fostered greater mutual empathy and improved the quality of the relationship. On the one hand, the partners modified their attributions, perceiving fewer malicious intentions in the gambling behavior. On the other hand, the gamblers were more aware how much their gambling behavior hurt their partners. These research results indicate that the partners' realized that the gamblers' poor behavior was not an indication of a deliberate wish to hurt the partners, thus making reconciliation easier (Hook et al., 2015). Gender-based differences have been noted where, in women, changes in attributions helped them to forgive their spouses, whereas in men, more empathy toward the wives increased the men's ability to forgive them (Fincham et al., 2002). It is not surprising that the theme of a change in attributions was present in the statements of couples who received couple treatment, since gamblers' excessive behavior is a good example of relationship trauma. Their behavior violates the partners' expectations and beliefs about an attachment relationship, and, in so doing, destabilizes the partners' feeling of security by undermining their ability to predict future behavior (Gordon and Baucom, 1998). The concept of forgiveness, used in several studies exploring relational trauma in couples (Greenberg et al., 2010), presents several similarities with the processes evoked by the participants, for example, the changing of negative feelings (anger, feelings of vengeance) into greater attachment and empathy (Malcolm et al., 2005) and the acquisition of a more realistic view of the spouses (Gordon and Baucom, 1998). It would seem worthwhile to further pursue research into the role of forgiveness in couples where one member is a gambler. Studies should focus on the therapeutic strategies that best facilitate this improvement in negative feelings, since this process of forgiveness is predominant in predicting relationship improvement after the treatment (Meneses and Greenberg, 2014). Furthermore, it would seem to be difficult to embark on a forgiveness process without outside help (Ferland et al., 2017).

Many of the participants in couple treatment reported on how the partners were able to help the gamblers avoid relapse. This observation contradicts those of another study conducted with gamblers and their partners who were not in treatment, the study revealing that most of them thought that the partners could not influence the gamblers' behavior (Tepperman and Korn, 2006). Several studies have noted that the partners sometimes had non-intentional behavior that favored gambling (Bertrand et al., 2008), one of these being relationship tensions associated with the partners' control of the money (Lorenz and Yaffee, 1989). As regards substance abuse, the partners were asked to do the following: increase behaviors that were identified as favouring abstinence (e.g., avoid exposure to substances or at-risk situations), to decrease those that inadvertently favored substance use (e.g., protect the addict from the natural negative consequences of substance use) and be actively available to talk about difficult situations or other aspects related to reduction (Meyers and Smith, 2009; O'Farrell and Clements, 2012). Clinical articles (McGurrin, 1992) and studies (Krishnan and Orford, 2002) that have focused on pathological gambling would seem to point in the same direction, noting that partners reimbursed debts, apologized to bosses for work absences, and took charge of negotiations with creditors, thereby potentially fostering continued gambling behavior. The statements of the participants in the present study, specifically those in couple treatment, tended to confirm these hypotheses, namely that partners can help gamblers stop gambling and avoid relapses. They likewise pointed out that the partners played a “supervisor” role in avoiding relapse and providing more enjoyable couple activities with no connection to gambling.

Several participants underlined the intrinsically relational aspect of gambling behavior problems, thus emphasizing the need for the partner to be involved in the treatment. This observation was probably at the basis of the participants' considerable satisfaction with the couple treatment and the interest of several of the participants in individual treatment to participate in the former. This emphasis on the relationship aspect in pathological gambling is commented on by numerous authors who highlight the considerably negative impact that gambling has on the partners and the family (Dickson-Swift et al., 2005; Ferland et al., 2008; Kourgiantakis et al., 2013). Gambling erodes trust and provokes depression and anxiety in the family (Ferland et al., 2017), and leads to less quality time spent together and poorer communication (Dowling et al., 2016; Ferland et al., 2017). This relationship dimension could also be seen in the benefits arising from involving family members (Ingle et al., 2008), in the pathological gamblers' treatment, particularly the partners (Lee and Awosoga, 2015). However, a few participants, primarily gamblers who had taken part in individual treatment, considered that the spouses were not concerned by what was discussed in treatment and that they would be wasting their time. These statements were similar to those made by gamblers who were not undergoing treatment as well as to those of their partners (Tepperman and Korn, 2006). It would thus seem that participating in couple treatment made gamblers realize the importance of the relationship dimension in their problem, and thus the need for couple treatment that takes this into account. Among gamblers who were not involved in treatment where the spouses participated, it was more likely that they mentioned that their partners' involvement was not necessary.

The problem of gamblers dropping out of treatment early has been described as a priority subject in clinical research (Toneatto and Millar, 2004). This being said, the gamblers who were oriented toward couple treatment reported how they went to treatment more regularly because of their partners' presence. Partners whose gambling spouses were oriented toward individual treatment mentioned how they would have liked to have had more influence on the gamblers' presence at appointments, and how this would have been easier in couple treatment. Some research results have shown that the fact of living together positively influenced presence in the treatment among pathological gamblers (Aragay et al., 2015), though other results did not confirm this observation (Ingle et al., 2008). It would seem nonetheless that continuing in treatment is more likely when there is support in the gamblers' environment (Grant et al., 2004). When family actively participated in the treatment, the pathological gamblers were present more often and remained 30% longer than those who had no family members involved (Ingle et al., 2008). Even direct but minimal help provided solely by concerned significant others helped to reduce the number of gambling days and improved the quality of the relationship (Hodgins et al., 2007).

Some participants expressed an interest in an approach that would combine individual and couple treatment. No clinical trials of this type are known to have been conducted in the treatment of pathological gambling. That being said, in substance abuse treatment, a combination of individual or group treatment on the one hand and couple treatment on the other proved to be more effective than individual treatment only (O'Farrell and Clements, 2012), which opens the door to a possible combination of these two treatment types. Conversely, couple treatment in a group setting proved to be clearly less effective than standard couple treatment (in the two cases in combination with individual treatment) in work with alcoholics, highlighting the importance of evaluating the effectiveness of various ways of providing treatment (O'Farrell et al., 2016).

The participants likewise remarked on conditions that favored or hindered their participation in one type of treatment or the other. Participants in couple therapy revealed the importance of having a satisfying couple relationship at the beginning of the therapy and the wish to preserve this relationship as factors that contributed to a successful couple therapy. These statements are supported by clinical and research literature that reports how relationship stability is a plus in couple treatment for addiction (O'Farrell and Fals-Stewart, 1999). Not surprisingly, the scientific literature also shows that all the indicators of greater problem severity are identified as being associated with less successful couple treatment for substance abuse. It is likely that this also applies to couple treatment for pathological gambling. Frequently reported signs are severe consumption of drugs and alcohol, drug and alcohol related problems in both members of the couple, severe and chronic violence (Birchler et al., 2008), and mental health problems (O'Farrell and Fals-Stewart, 2006; Birchler et al., 2008). Moreover, some of the participants stated that, if the gamblers did not wish to tell their partners about all their problems, it would be better to use individual therapy or a combination of the two. Future research should try to establish criteria for directing gamblers who are in relationships toward individual, couple, or combined treatment.

The present study has some limitations. To begin with, it is reasonable to think that the people who agreed to participate in the study had a favorable attitude toward involvement of the couple in treatment, since this was one of the two types of treatment in which they would take part. It is likely that relationship issues were at the heart of the concerns of gamblers and their partners who accepted to participate in the project and that their comments would be more favorable toward couple treatment. A social desirability bias may thus have been present. Furthermore, even though this study was presented neutrally as a comparison of the two treatment types, it is possible that the participants had the impression that the researchers and clinicians favored couple treatment, and that they subsequently wanted to please the research team in their answers during the qualitative interview. Likewise, it would have been beneficial to question people pre-treatment as to their impressions about the pros and cons of each type, and then compare post-treatment their subsequent opinions about the treatment and its impact on their rehabilitation. Contrast analyses as a function of gender, the presence or not of children, and the seriousness of the gambling problems would have been interesting to explore, but the sample size was too small to permit such analyses.

## Conclusion

The participants brought up several relationship issues that were associated with the fact that one member of the couple was a pathological gambler, and consequently insisted on how couple treatment helped them to deal with these issues and their overall situation. These statements were made by both the gamblers and partners. They were proportionally more frequent—indeed, practically unanimous—among the participants in the couple treatment, but were also made by several participants in the individual treatment group who perceived several potential benefits in the couple treatment. Conversely, the advantages of individual treatment were almost exclusively emphasized by people who partook in this treatment, which suggests that the disadvantages of the couple treatment stemmed more often from anticipatory fear rather than from actual experiences. That being said, the individual treatment did seem to have some undeniable benefits.

The participants' statements highlighted the interest and benefits of couple treatment and support the clinical relevance. The couples, one of whom was a pathological gambler, had to learn to mutually understand each other and partake in the reconstruction of their trust. For this, they needed outside help. That said, the partners also became a source of aid during this process. These results highlight the necessity to pursue research into how couples change when one member tries to put an end to pathological gambling. For example, the dimension of forgiveness but also the trauma experienced by the partners would be worthy of special attention in future research, in couple treatment but also in individual treatment for the partners. The current results likewise highlight the necessity to evaluate the pertinence of the various types of treatment for gambling, including the different ways of involving family members. It is also worth noting the need to pursue research with clientele with concomitant problems related to substance abuse and gambling. Clinical practitioners participating in the study pointed to the regular frequency of these concomitant problems. In short, a range of treatment targeting the multiple needs of gamblers and their partners would seem to be a response that is better adapted to a complex problem like pathological gambling. Future research should look for different ways of integrating partners into the treatment.

## Author contributions

JT: Co-contributions to the conception or design of the work. Co-interpretation of data for the work. Co-drafting the last version. Final approval of the version to be published. MD, KB, NB-M, FF, A-CS, MS-J: Co-contributions to the conception or design of the work and revisiting and critically the drafting. MC: Analysis and co-interpretation of data for the work. Drafting the first version. Validate references.

### Conflict of interest statement

The authors declare that the research was conducted in the absence of any commercial or financial relationships that could be construed as a potential conflict of interest.

## Footnotes

^1^In the present article, the terms *gambler, pathological gambler, spouse*, and *gambling spouse* all refer to the same member of the couple, namely the person with the pathological gambling problem. For most part, this person was male, and thus masculine pronouns are used in this article to refer to this person. Conversely, the term *partner* refers to the non-gambling member of the couple, for the most part female. Pronoun gender follows accordingly.

^2^This study was approved by three ethics committees: the Comité d'éthique de la recherche-Dépendance-inégalités Sociales et Santé Publique (DIS) of the CIUSSS of the Centre-Sud-de-l'île-de-Montréal (CÉRT-2010-112), by the ethics committee of the Université du Québec à Trois-Rivières (CER-10-156-06.13), and by the Comité d'éthique de la recherche en santé chez l'humain du Centre Hospitalier Universitaire de Sherbrooke (CHUS) (2011-284, 10-171).

^3^But not during the last 6 months before the study started.

^4^Each interview verbatim citation is identified by (a) a unique number to each individual, (b) the status of the participant as a gambler or a partner, and (c) the modality of treatment received (individual therapy, couple therapy or even both for a few exceptions).
